# Ultra-wideband optically transparent flexible metamaterial absorber for satellite stealth

**DOI:** 10.1038/s41598-025-09951-7

**Published:** 2025-08-08

**Authors:** Chao Zhao, Min Jia, Ningtao Zhang, Shiyao Meng, Yanhong Tian

**Affiliations:** 1 Communication Research Center, School of Electronics and Information Engineering, Harbin Institute of Technology, Harbin, 150001 China; 2 State Key Laboratory of Precision Welding & Joining of Materials and Structures, Harbin Institute of Technology, Harbin, 150001 China

**Keywords:** Flexible metamaterial, Broadband absorber, Optical transparent, Polarization insensitive, Satellite stealth, Engineering, Materials science, Optics and photonics, Physics

## Abstract

With the rapid advancement of space technology, the stealth capabilities of satellites have become crucial for enhancing their survivability and increasing mission success rates. However, traditional absorbing materials do not adequately address the requirements for flexible conformal designs, high transmittance in the visible light spectrum, and ultra-wideband absorption capabilities in microwave frequencies. To address this issue, we developed an ultra-thin, flexible, ultra-wideband metamaterial absorber that is optically transparent and insensitive to polarization and angle. This design is based on indium tin oxide (ITO) material. The results of the electromagnetic simulation indicate that within the frequency range of 11.09 GHz to 108.20 GHz, the absorption rate of the absorber exceeds 90%. The total absorption bandwidth is measured at 97.11 GHz, resulting in a relative absorption bandwidth of 162.81%. In addition, it continues to demonstrate effective absorption performance for electromagnetic waves with an incident angle of less than 45°. The absorber designed in this study exhibits polarization-insensitive characteristics, enabling it to effectively address the detection of multi-polarization radar. Furthermore, it is compatible with the arbitrary deployment attitudes of the carrier, which significantly enhances the operational reliability of the stealth system in actual combat scenarios.

## Introduction

 With the rapid advancement of sophisticated military technologies, detection and tracking capabilities across various nations have become increasingly refined. This evolution presents a significant threat to the survivability of satellites and fighter jets, while simultaneously imposing more stringent requirements for the concealment of critical military equipment^1–4^. To address this challenge, electromagnetic stealth technology has emerged as a focal point for numerous research teams both domestically and internationally^5–8^. Traditional wave-absorbing materials face several challenges, including a limited absorption bandwidth and inadequate flexibility, which restrict their practical applications. In contrast, metamaterials exhibit remarkable physical properties and can be engineered into artificial composite structures^9^. Stealth technology utilizing metamaterials has garnered significant attention from researchers due to its simple structure, lightweight design, high degree of design flexibility, and the ability to effectively manipulate electromagnetic waves^10,11^. Over the past decade, this field has experienced rapid development. As a promising alternative to conventional wave-absorbing materials, metamaterial wave absorbers play a crucial role in stealth applications across various scenarios^12–15^. In recent years, the demand for ultra-wideband metamaterial absorbers has become increasingly pressing. Various methods have been employed to broaden the absorption band, including resonant structures^16^ loading active devices^17,18^ and utilizing multilayer stacking^19^. Additionally, UWB absorbers have been designed using materials with high dielectric loss^20,21^. However, these approaches generally result in increased thickness and manufacturing complexity.

Traditional electromagnetic metamaterials typically employ thick and rigid structures. In recent years, significant advancements have been made in the design of metamaterial absorbers within the K-band. The research team demonstrated a wide absorption bandwidth and directional response to both normal and oblique incidence by employing a symmetrically arranged structure. This broadband, wide-angle, and direction-independent characteristic greatly enhances the performance of the absorber^22,23^. However, its metallic composition limits its conformability with equipment such as satellites and unmanned aerial vehicles. The aforementioned wave absorbers present several challenges in the context of satellite stealth: (1) Metal coatings obstruct optical sensors. (2) The rigid substrate hinders the deployment of solar wings. (3) They cannot conform to the satellite’s structure. (4) There is a lack of optical transparency, preventing the satellite’s solar wings from effectively harnessing solar energy.

Consequently, the development of flexible electromagnetic metamaterial wave absorbers has become increasingly significant^24^. Common materials utilized for the fabrication of flexible layers include polydimethylsiloxane (PDMS), polyimide (PI), polyethylene terephthalate (PET), and polymethyl methacrylate (PMMA)^25,26^. If the medium that conforms to the surface of the object is utilized as the intermediate layer of the wave absorber, it enables the wave absorber to be bent and applied to non-planar objects. This innovation significantly broadens the application range of metamaterial wave absorbers^27,28^. In recent years, the rapid increase in application demand has led to significant attention being directed towards the research of transparent wideband absorbers.

The design of transparent materials, along with the introduction of artificial electromagnetic metamaterials, has led to significant advancements in the research of transparent wideband metamaterial absorbers^29–34^. Satellite stealth technology serves a distinctive function within space attack and defense systems, while solar panels are essential energy devices utilized on satellites. However, the pronounced backscatter characteristics of metals used in solar panels pose a significant threat to the safety and survivability of satellites. Traditional coated absorbent materials do not ensure efficient optical transmittance during the operation of solar panels^35,37^. The advent of optically transparent metamaterial absorbers has introduced a novel approach to the microwave stealth design of solar panels^39–41^. However, simultaneously achieving flexible conformality, high visible light transmittance, and broadband microwave absorption remains a significant challenge in the design of wave absorbers^42,43^.

In order to solve the above requirements, this paper proposes a flexible metamaterial with low profile, small size and high transparency of “conductive film-dielectric layer-conductive film” structure by optimizing the design of resonant layer pattern based on impedance matching principle. The periodic dimensions of the structural element proposed in this paper measure only $p{\text{ = 8 mm}}$, with a total thickness of approximately $0.75{\text{ mm}}$. This design effectively addresses the issues associated with the large size and thickness commonly found in existing wave absorbers. The results of the electromagnetic simulation indicate that within the frequency range of 11.09 GHz to 108.20 GHz, the absorber exhibits an absorption rate exceeding 90%. The total absorption bandwidth is measured at 97.11 GHz, resulting in a relative absorption bandwidth of 162.81%. After conducting electromagnetic simulations at various incidence angles, the results demonstrate a commendable degree of polarization insensitivity and stability in absorption under oblique incidence conditions. In addition, the mechanism of wideband absorption is investigated through an analysis of the surface current, power loss density, and energy distribution density of the electromagnetic field. The flexible transparent absorber examined in this study, characterized by its high absorption efficiency, minimal thickness, compact size, lightweight design, and broad absorption frequency range, offers innovative solutions for future communication technologies and electronic devices. Its significance for practical applications is substantial.

This work provides a core component design method for the next-generation intelligent stealth satellite, featuring ultra-low profile, wide bandwidth, polarization and Angle insensitivity, as well as optical transparency. This work can simultaneously ensure its good working efficiency and microwave stealth when applied to solar panels. This work can be extended to the terahertz frequency band in the future or dynamic reconfigurable materials (such as liquid crystals and phase change materials) can be introduced to further enhance environmental adaptability.

## Analysis of broadband absorbers model

Electromagnetic waves incident upon the surface of an absorber exhibits a dual behavior: a portion of the electromagnetic wave is reflected, while another portion penetrates the absorber and continues to propagate. The absorption performance of a wave absorber can be quantified by its absorption rate $A(\omega )$, which is related to both reflectivity $R(\omega )$ and transmittance $T(\omega )$. This relationship can be expressed as follows:

1 $$A(\omega )=1 - R(\omega ) - T(\omega )=1 - {\left| {{S_{11}}} \right|^2} - {\left| {{S_{21}}} \right|^2}$$

${S_{11}}$ and ${S_{21}}$represent the reflection coefficient and transmission coefficient of the electric field respectively. In the absorber designed in this paper, the back layer is completely covered by the good conductor material ITO, so the transmittance can be simplified as follows:

2 $$A(\omega )=1 - {\left| {{S_{11}}} \right|^2}$$

The input impedance of the absorber is ${Z_{{\text{in}}}}$, then the reflection coefficient can be expressed as:

3 $${S_{11}}=\frac{{{Z_{{\text{in}}}} - {Z_0}}}{{{Z_{{\text{in}}}}+{Z_0}}}$$

Where ${Z_0}=377{\text{ }}\Omega$ is the characteristic impedance of free space, and the input impedance of metamaterial absorber is ${Z_{{\text{in}}}}$.

4 $${Z_0}=\sqrt {\frac{{{\mu _0}}}{{{\varepsilon _0}}}} =377{\text{\varvec{\Omega}}}$$

5 $${Z_{{\text{in}}}}=\sqrt {\frac{{{\mu _0}{\mu _{\text{r}}}}}{{{\varepsilon _0}{\varepsilon _{\text{r}}}}}}$$

The objective of the absorber design is to optimize the parameters of the unit structure in order to achieve an impedance match as closely as possible, thereby attaining a reflectivity of 0. This approach aims to approximate the ideal absorption effect of 1.

### Ultrawideband absorber structure design

**Fig. 1:**
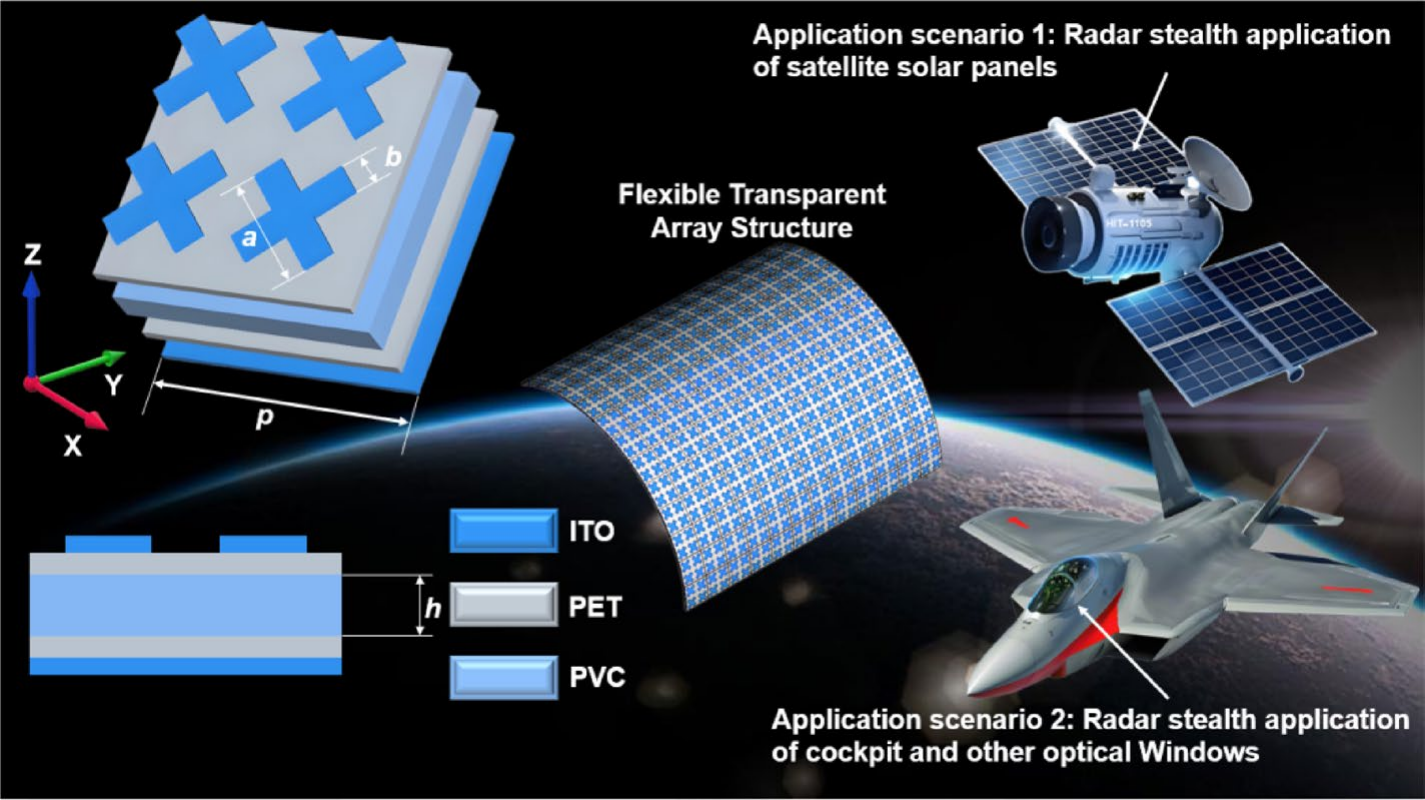
Structure diagram of metamaterial absorber and its main application scenarios. $p{\text{ = 8 mm}}$ , $h{\text{ = 0}}{\text{.5 mm}}$, $a{\text{ = 3}}{\text{.5 mm}}$, $b{\text{ = 0}}{\text{.9 mm}}$.

As illustrated in Fig. 1, this paper presents an ultra-wideband flexible transparent ultra-thin metamaterial absorber. The design comprises a periodic sequential arrangement of unit structures, each with a side length of. The dielectric substrate is PVC whose relative dielectric constant is 3, loss Angle tangent value is 0.014, and thickness is. The reflective substrate consists of a PET base entirely coated with an ITO film, exhibiting a square resistance of. Among the materials considered, the thickness of the PET substrate is, with a relative dielectric constant of 3 and a loss tangent value of 0.06. From an engineering implementation perspective, it is common for PET thicknesses to be either or. The uppermost layer consists of four resonant units arranged in a cross pattern, constructed from ITO film with a square resistance of.

The metamaterial absorber presented in this paper demonstrates significant potential for applications in satellite solar panel stealth and radar stealth for optical windows, such as those found in fighter cockpits. The CST Studio Suite 2021 simulation software was employed for modeling and simulation purposes. The boundary conditions in the x and y directions were defined as “unit cell,” while the z direction was designated as an open boundary. In the simulation, the wave source type is a plane wave, and the background space should be a uniform normal material. It was assumed that the electromagnetic wave would incident along the negative direction of the z-axis. To achieve optimal absorption performance, simulation software is employed to analyze and refine the relevant parameters. The optimized values for each parameter are presented in Table 1.

**Table 1:** Parameter size of optimized absorber.

Parameters	Size/mm
a	3.5
b	0.9
h	0.5
d	0.125

A satellite represents a complex systems engineering project that encompasses numerous subsystems, including measurement and control, structural integrity, thermal management, power supply, data handling, and attitude control. Stealth technology functions as a critical subsystem that must integrate seamlessly with all other subsystems. During the in-orbit operation of satellites, they encounter extreme environmental conditions such as ultraviolet radiation, microgravity, and high vacuum. These factors can exert corrosive and degrading effects on the surface materials of the satellites. Consequently, it is imperative for stealth materials to exhibit robust resistance to these harsh space environments. The average temperature in space is approximately − 270.3 °C. However, due to solar radiation exposure along with the satellite’s own power consumption and thermal control measures, temperatures on the star’s surface and within solar cell arrays can fluctuate between − 90 °C to + 120 °C. This variability imposes stringent requirements regarding the operational temperature range of stealth materials. Furthermore, mission specifications necessitate that stealth satellites possess long-duration orbital capabilities; thus making repair or replacement of stealth materials exceedingly challenging once deployed. As a result, there exists a significant demand for stealth materials designed for extended service life in satellite applications.

ITO exhibits high electrical conductivity and visible light transmittance. When integrated with metasurface design, it can achieve over 90% broadband absorption while preserving optical transparency. PET, as a flexible substrate, supports ITO films in forming lightweight and conformal structures that are well-suited for the stealth requirements of curved satellite surfaces. However, the conductivity of ITO may be compromised by high-energy particle radiation; thus, enhancing its radiation resistance through surface coating is essential. The ultraviolet stability of PET is relatively inadequate, necessitating the incorporation of an anti-UV coating or the utilization of modified PET materials. Currently, significant challenges include the upper limit of temperature resistance and long-term radiation aging of PET; therefore, further implementation of an active temperature control system for satellites is required.

## Results and discussion

**Fig. 2:**
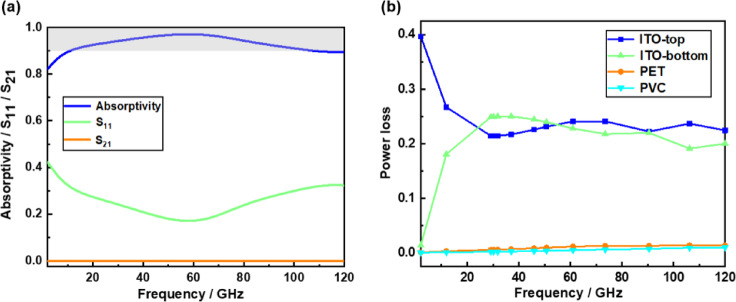
Simulation results of absorptivity, S parameter, normalized impedance and power loss of the proposed absorber.

After conducting the simulation, the curves depicting the absorption rate, reflection coefficient${S_{11}}$, and transmission coefficient ${S_{21}}$ as functions of frequency are presented in Fig. 2 (a). It is evident that the transmission coefficient approaches zero. This phenomenon can be attributed to the use of an ITO conductive film with low square resistance as the reflective substrate at the bottom, which facilitates total reflection.

In the frequency range of 11.09 GHz to 108.20 GHz, the absorber exhibits an absorption rate exceeding 90%. Furthermore, the relative absorption bandwidth can attain a remarkable value of 162.81%, thereby achieving ultra-wideband perfect absorption. According to the equivalent medium theory, the findings demonstrate that the designed absorber exhibits outstanding impedance matching characteristics with free space throughout its entire operational bandwidth. Consequently, electromagnetic waves can be absorbed nearly in their entirety. In addition, Fig. 2 (b) presents the simulated power loss of each material in response to the incident wave. The calculation results indicate that the ITO conductive film with high square resistance at the top is the primary contributor to electromagnetic wave absorption, followed by the ITO conductive film with low square resistance at the bottom. In contrast, both the PET substrate and PVC dielectric layer exhibit minimal consumption of electromagnetic waves.

The fundamental working principle of the metamaterial absorber presented in this paper is based on impedance matching and resonance loss, rather than predominantly relying on the intrinsic losses of the substrate material. The design objective is to minimize reflection by meticulously regulating the geometric parameters of the surface ITO pattern (acting as a resonant structure) and the intermediate dielectric layer (PET), including factors such as unit size, period, thickness, etc. This approach aims to ensure that the equivalent input impedance of the metamaterial surface closely approximates that of free space.

Meanwhile, the designed resonance mode (plasma resonance) effectively confines the incident electromagnetic wave energy within the structure, particularly concentrating it in the high-loss ITO layer and PET dielectric layer, thereby converting it into thermal energy. As a consumable material, ITO films serve as a crucial mechanism for ohmic loss across the designated frequency band. The square resistance value has been optimized to ensure that it not only provides adequate conductivity to support resonance but also generates significant resistive losses to dissipate electromagnetic energy. The ITO layer is one of the primary contributors to high-frequency loss in the absorber, and its role has been prioritized and maximized in this design framework. Under this design paradigm, the substrate (PVC) primarily functions to provide mechanical support and establish a basic dielectric environment for the entire flexible structure rather than acting as a principal source of loss. The absorption performance is predominantly governed by the electromagnetic response of both the top ITO resonator and the intermediate PET layer.

The thickness of the PVC substrate ($h{\text{ = 0}}{\text{.5 mm}}$) is significantly smaller at a higher frequency of 100 GHz (with a wavelength ${\lambda _0}{\text{ = 3 mm}}$). This reduction in thickness limits the path length of electromagnetic waves propagating within the PVC substrate, thereby substantially decreasing its overall loss contribution. Thicker substrates would exacerbate the loss issue; however, our design has opted for relatively thin substrates that fulfill the requirements for flexibility, mechanical strength, and optical transparency.

**Fig. 3:**
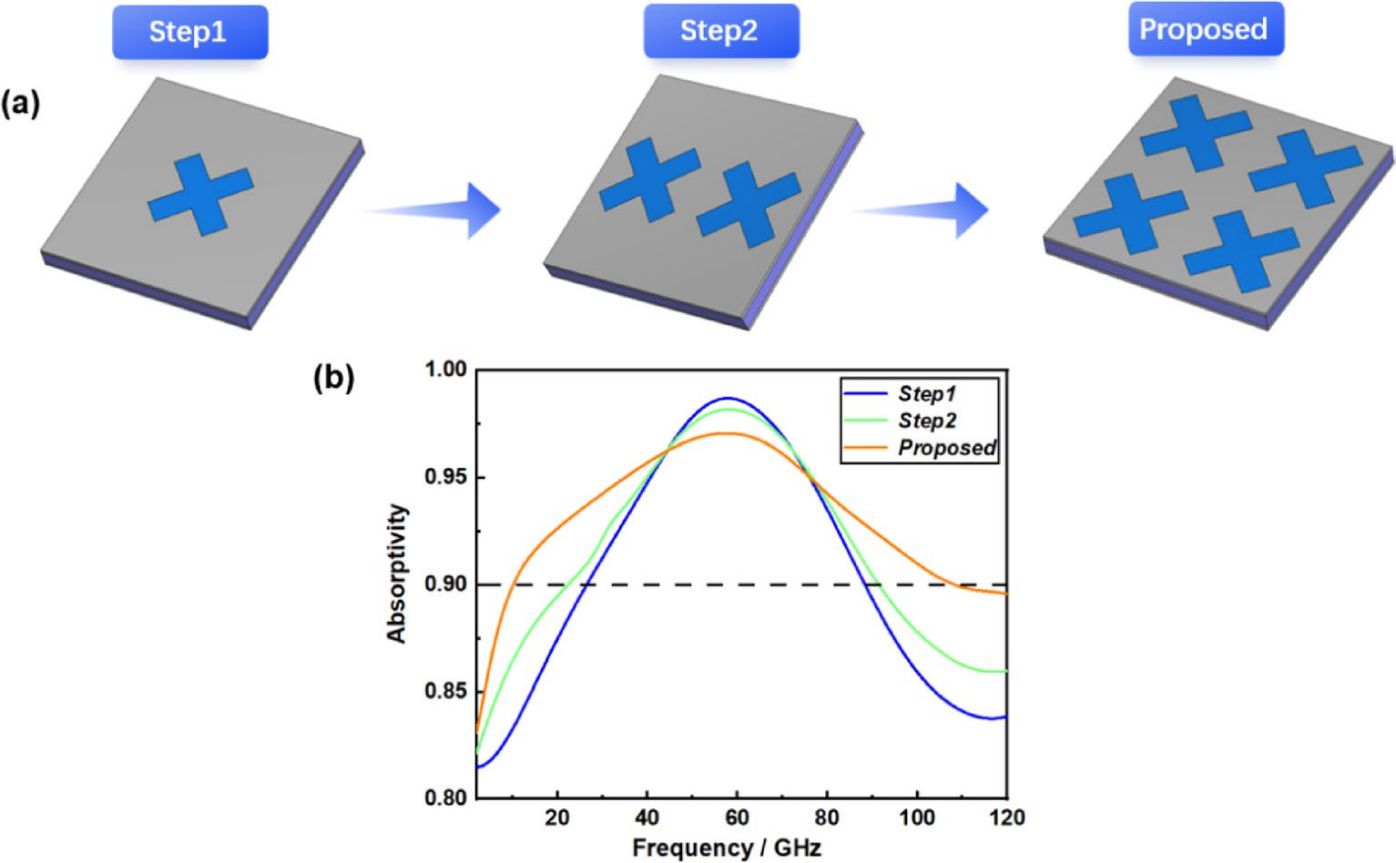
Design process of metamaterial absorber. (a) Design steps. (b) Absorption corresponding to each step.

As shown in Fig. 3, to demonstrate the absorption effectiveness of the designed resonant layer structure, we first developed an indium tin oxide (ITO) film configured as a conventional cross structure (Step 1), achieving an absorption rate exceeding 90% within the frequency range of 26.4 GHz to 88.4 GHz.

Subsequently, in Step 2, we modified the top layer of the ITO film to incorporate two symmetrical cross structures positioned on either side. This modification resulted in a broadened absorption bandwidth, with an absorption rate also surpassing 90% across the frequency range of 22.3 GHz to 91.4 GHz. Finally, we proposed a design for the top layer of the ITO film featuring an axially symmetrical four-cross structure. It is evident that increasing the number of cross-shaped structures enhances resonance between electromagnetic waves and the conductive film. The analysis presented above indicates that our patterned ITO thin-film absorber exhibits exceptional absorption characteristics.

**Fig. 4:**
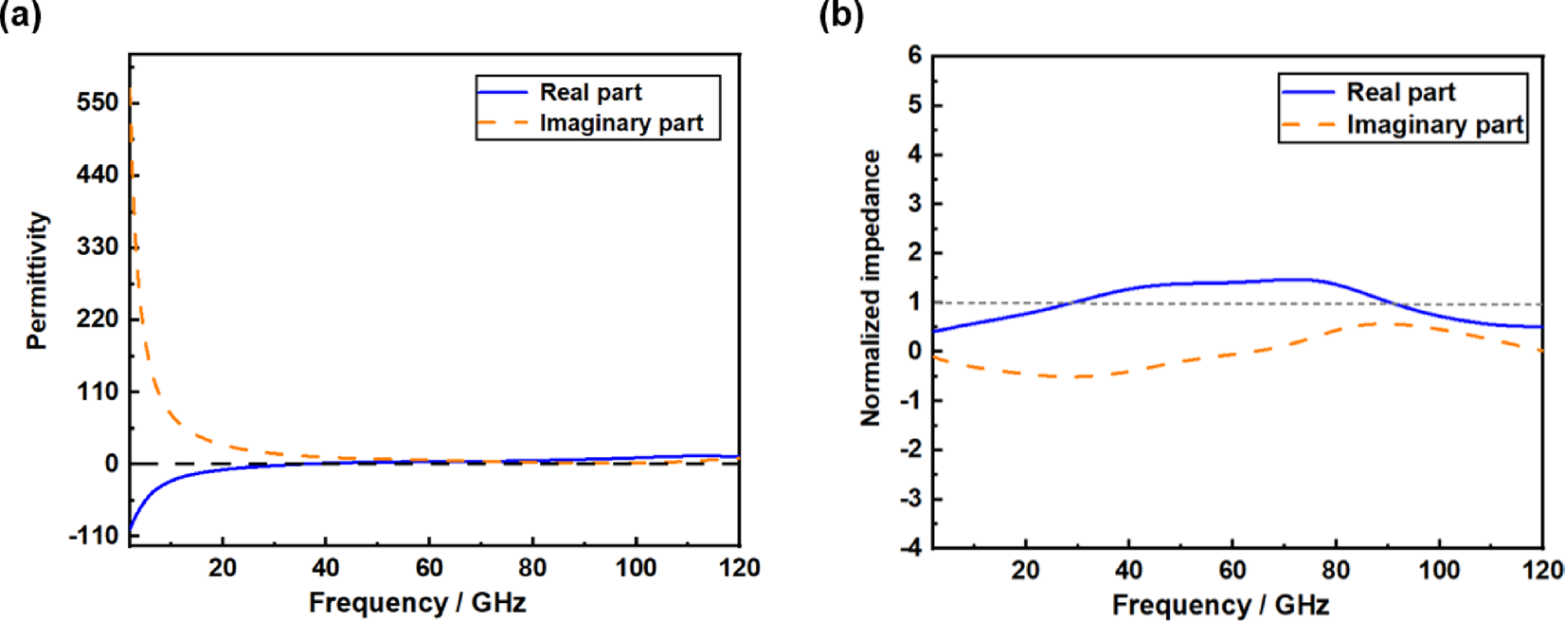
(a) The permittivity of ITO in the investigated frequency band. (b) Normalized impedance of proposed absorber.

As shown in Fig. 4(a), the real and imaginary parts of the complex dielectric constant of the metamaterial absorber are presented. As shown in Fig. 4 (b), within the absorption frequency band, the real part of the normalized impedance of the absorber is close to 1, and the imaginary part is close to 0. This result indicates that within the entire working frequency band, the designed absorber has good impedance matching characteristics with the free space, and the electromagnetic waves can be almost completely absorbed.

**Fig. 5:**
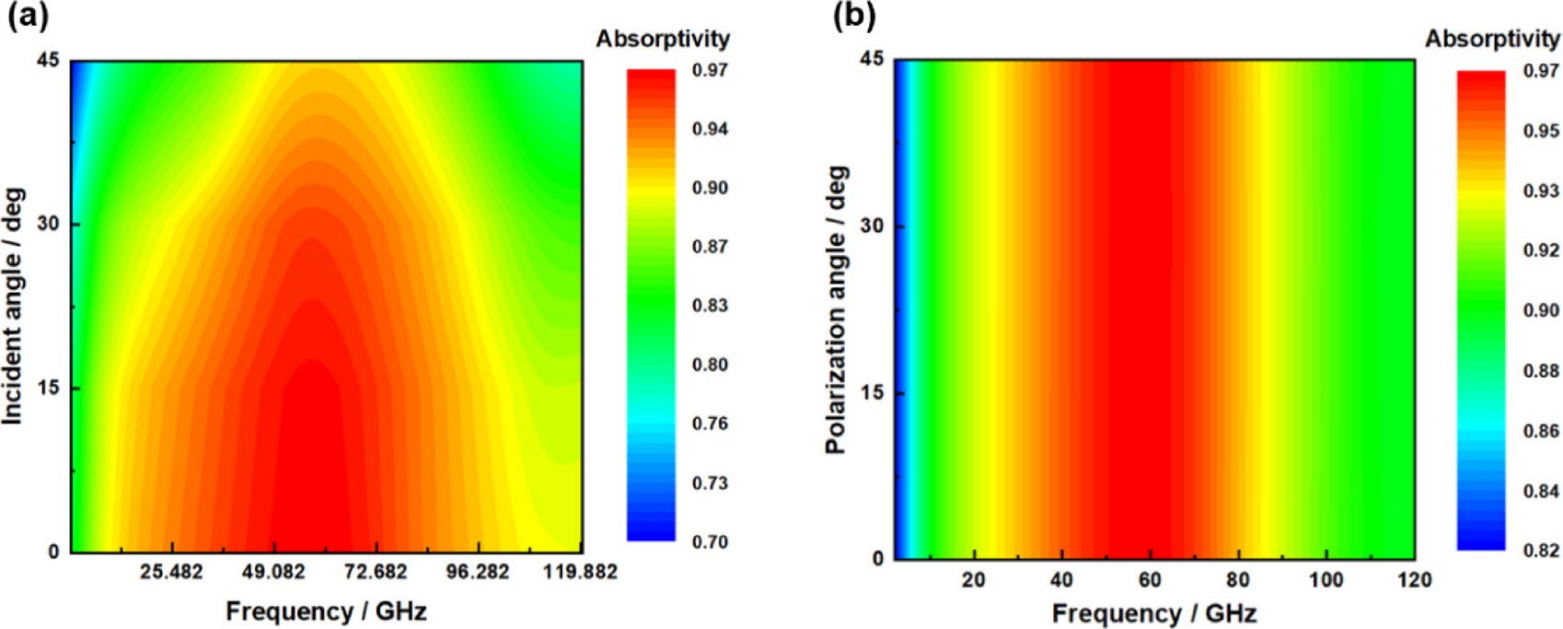
Absorptivity at different incident angles and polarization angles.

The angular stability serves as a fundamental index for the engineering application of absorbers, directly influencing their stealth reliability within complex electromagnetic environments. When the incident wave deviates from the normal direction, traditional absorbers experience a significant reduction in absorption rate due to impedance mismatch, resulting in a marked increase in the radar scattering cross section (RCS) of the target. Especially in the multi-attitude satellite operation, the wide Angle incidence coverage directly affects the stealth efficiency. In addition, the distortion of local incident angles and multipath reflections induced by the configuration of the carrier surface necessitate precise control over subwavelength structures to ensure phase consistency across a wide range of angles.

Therefore, overcoming the bottleneck of angular stability is a crucial prerequisite for achieving all-weather electromagnetic stealth. In this study, we further investigate the impact of the oblique incidence angle of incident electromagnetic waves on their absorption properties. Figure 5(a) illustrates the absorption rate of TE waves at oblique incidence. As the angle of incidence increases, the absorption frequency band shifts towards higher frequencies, while the absorption rate gradually decreases. Notably, when the angle of incidence is less than 45°, a broadband absorption performance with an absorption rate exceeding 75% can still be maintained over a relatively wide range.

One of the key indicators for assessing the absorption performance of a designed absorber is whether stable absorption can be maintained across different polarization angles. As illustrated in Fig. 5 (b), the absorption curve exhibits a near coincidence as the polarization angle increases from 0° to 45° in increments of 15°. This phenomenon can be attributed to the complete centrosymmetric of the resonant unit pattern on the top layer, which results in a consistent absorption rate regardless of variations in polarization angle. This characteristic demonstrates excellent insensitivity to polarization.

Polarization insensitivity is a critical technical parameter for absorbers to effectively adapt to complex electromagnetic environments. In scenarios involving high-speed satellite motion and multi-path reflections, the polarization state of the incident wave exhibits time-varying randomness. This characteristic not only enhances compatibility with multi-polarization radar detection but also accommodates any attitude deployment of the carrier. Consequently, it significantly improves the operational reliability of stealth systems in actual combat situations.

**Fig. 6:**
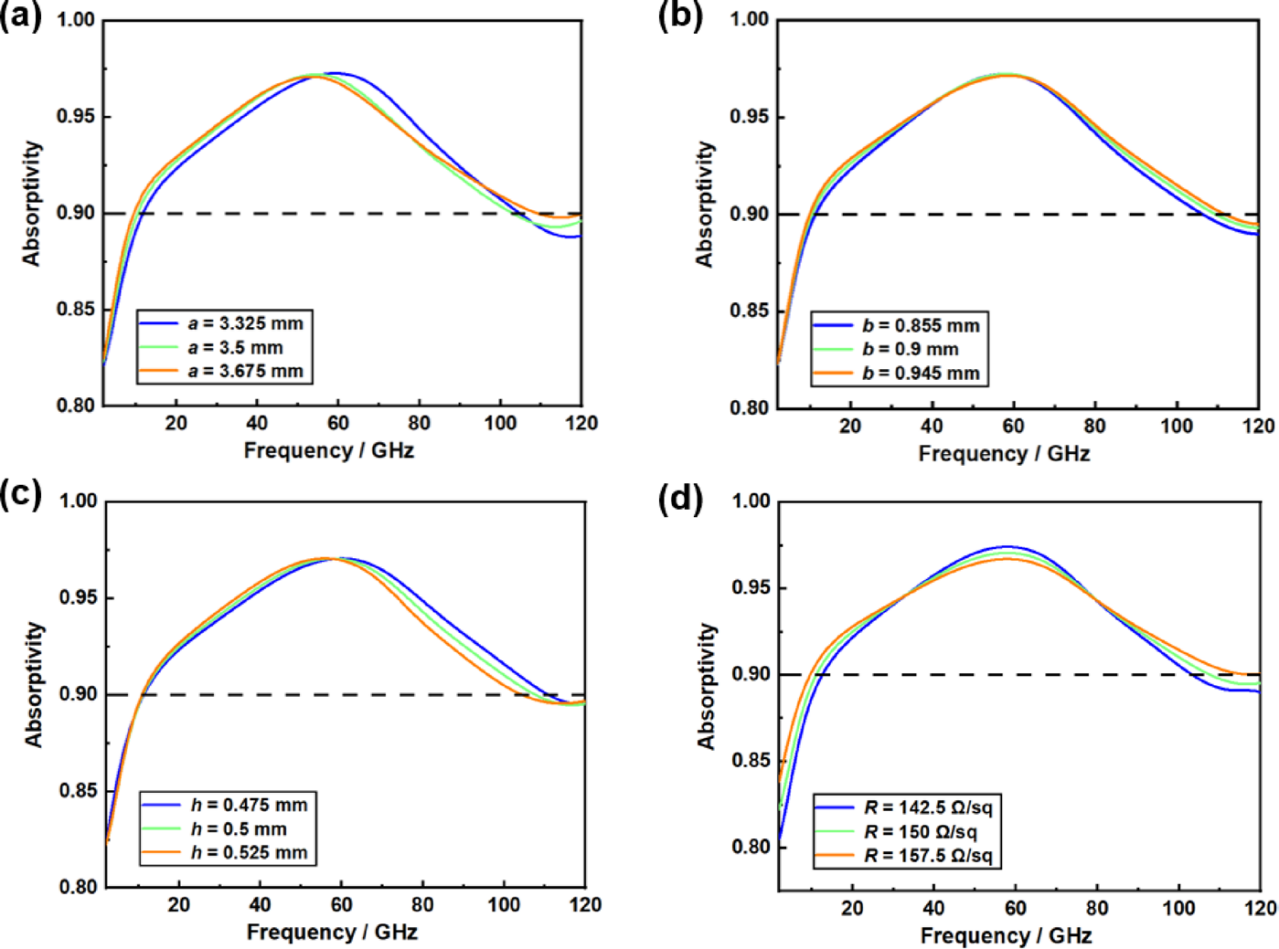
Absorptivity with ± 5% variation of metamaterial absorber geometric parameters.

To assess the impact of potential systematic geometric parameter errors that may arise during the actual machining of the designed structure, a robustness analysis of the geometric parameters is conducted. Based on the optimal design parameters, a variation of all geometric parameters (± 5%) is introduced to simulate potential errors that may occur during actual machining processes. It is evident from Fig. 6 that slight variations in certain geometric parameters of the structure have minimal impact on the absorption performance of the wave absorber. This observation underscores the robustness of the designed structure for practical engineering applications. The advancement in the durability of the absorber structure holds dual significance for engineering applications. First, from the perspective of manufacturing, geometric deformations resulting from micro and nano machining tolerances can lead to deviations in equivalent electromagnetic parameters. However, robust design methodologies can effectively manage the mismatching losses of the unit structure within a specified range, thereby enhancing the overall pass rate. Second, at the service level, structural deformation resulting from material expansion due to thermal cycling in space and mechanical vibrations can disrupt the conditions necessary for electromagnetic wave interference. A robust structure can mitigate these effects by establishing a tolerance region that maintains fluctuations in absorption rates within a specified range, thereby significantly extending the operational lifespan of the orbit. The enhanced robustness offers a dependable assurance for the broadband stealth capabilities of the spaceborne platform when operating under complex conditions.

**Fig. 7:**
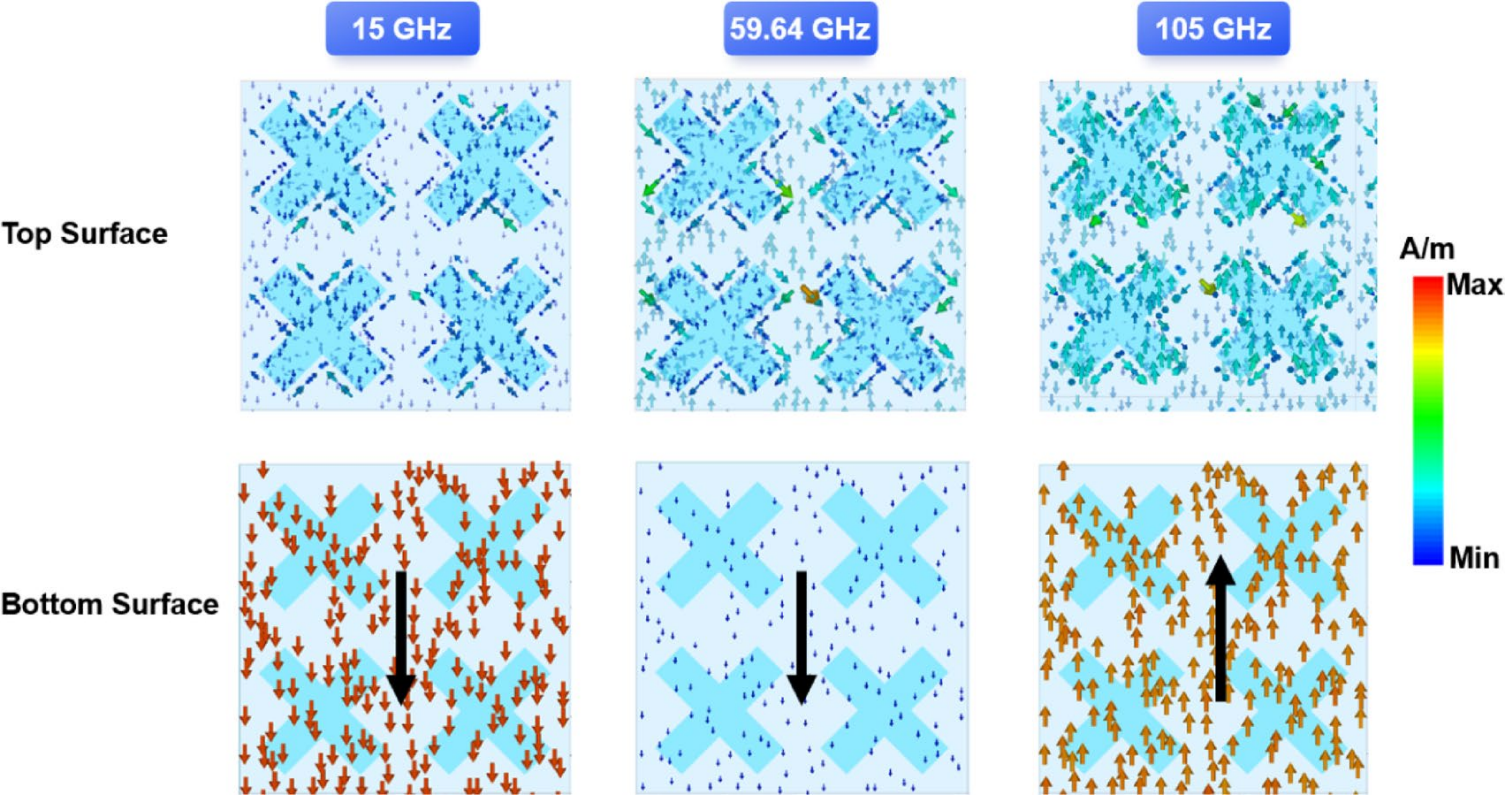
Surface current distribution at the resonant points and central frequency point.

To investigate the physical mechanisms underlying wideband absorption, surface current monitors were positioned at three specific frequency points: 15 GHz (low frequency), 59.64 GHz (central frequency), and 105 GHz (high frequency). This setup allowed for the observation of surface current distribution across these frequencies. Figure 7 illustrates the distribution of surface currents at three distinct frequency points, all presented on a uniform scale. At a frequency of 15 GHz, a clockwise current loop is induced to the left of the cross structure, while a counterclockwise current loop is generated to the right. At a frequency of 105 GHz, a counterclockwise current loop is induced to the left of the cross structure, while a clockwise current loop is generated to the right. The current flow of the top surface at the center frequency point is the same as that at the high frequency point. The broad absorption characteristics of the absorber can be attributed to the plasmon resonance generated by the charge carriers in the upper conductive film.

**Fig. 8:**
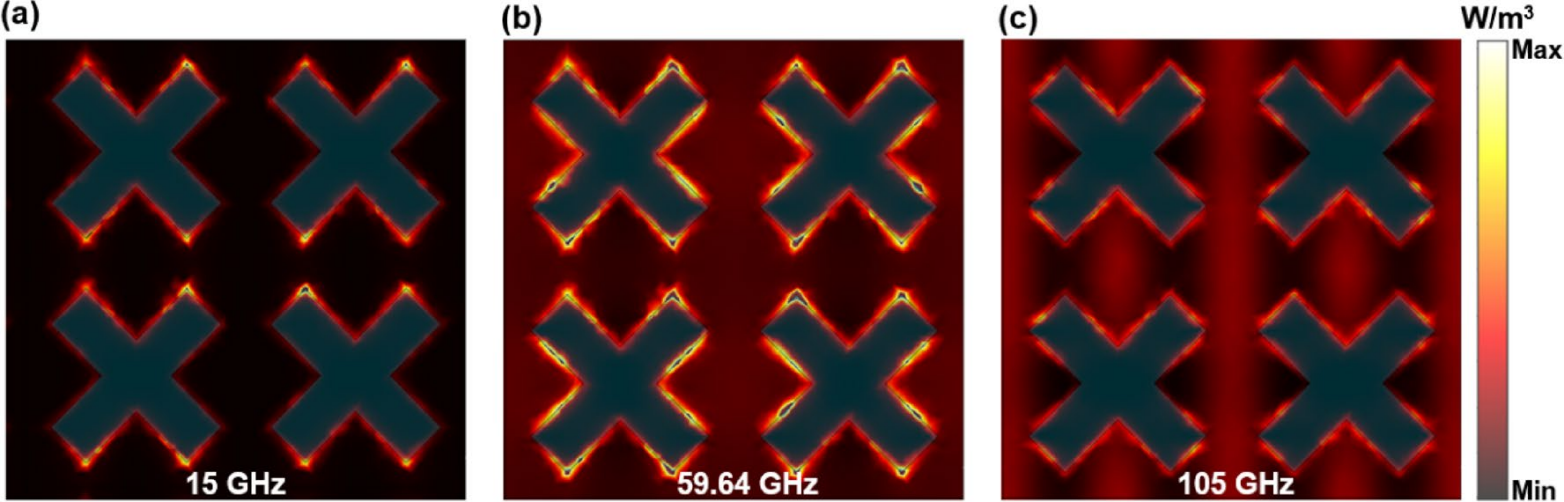
Distribution of power loss density at the resonant points and central frequency point.

According to the analysis in Fig. 2 (b), the top ITO conductive film is the main material that consumes electromagnetic waves. Therefore, the field distribution of the top ITO conductive film is further analyzed to further explore the physical mechanism of its wideband absorption. The distribution of power loss density, electric field energy density, and magnetic field energy density was observed by placing a field monitor at three distinct frequency points. It can be observed from Fig. 8 that the power loss density at the low frequency point of 15 GHz is most pronounced at both ends of the upper and lower sections of the cross structure. At the high-frequency resonant point of 105 GHz, the power loss density is predominantly concentrated at the cross edge. The density of power loss is maximized at the center frequency.

**Fig. 9:**
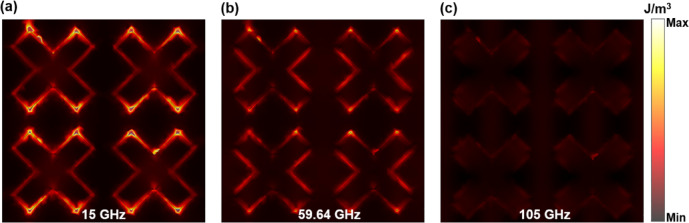
Distribution of electric field energy density at the resonant points and central frequency point.

**Fig. 10:**
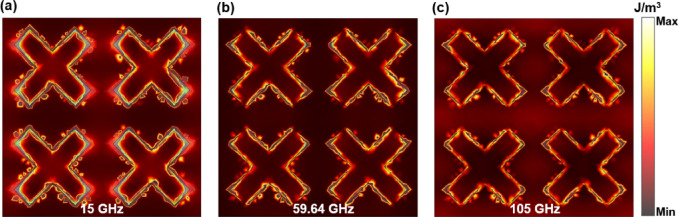
Distribution of magnetic field energy density at the resonant points and central frequency point.

As illustrated in Fig. 9, the energy density of the electric field at low frequencies exhibits a significant increase. The electromagnetic field is notably concentrated at both ends of the upper and lower sections of the cross structure, allowing for effective absorption and conversion of energy into Joule heat. As illustrated by the magnetic field energy density distribution in Fig. 10, the magnetic field energy is predominantly concentrated in the peripheral regions of the ITO structure. On the same scale, the energy of the magnetic field exceeds that of the electric field, indicating that magnetic resonance predominantly governs the behavior of the wave absorber. The electric resonance and magnetic resonance phenomena exhibited by the absorbing body facilitate the mutual conversion of energy within the electromagnetic field. When the equivalent permittivity and permeability of the two resonant excitations are closely matched, impedance matching in the metamaterial absorber is achieved, resulting in enhanced broadband absorption.

**Table 2:** Comparison of performance parameters between this work and other similar designs.

References	Absorption > 90%, Frequency band/GHz	Relative bandwidth	Thickness/mm	Flexible
^15^	6.9 ~ 22.7	106.8%	5.5	No
^29^	6.54 ~ 18.66	96.2%	5.35	Yes
^30^	8.02 ~ 33.91	123.5%	4.35	Yes
^31^	8.52 ~ 16.98	66.4%	3.325	Yes
^32^	8.0 ~ 32.0	120%	3.3	No
^37^	8.22 ~ 22.76	93.9%	2.85	Yes
^38^	13.30 ~ 39.76	99.7%	1.75	Yes
This Work	12.02 ~ 102.91	158.1%	0.75	Yes

Finally, this study compares several reported metamaterial wave absorbers based on conductive films with the wave absorbers proposed herein. The comparison is conducted in terms of frequency range with absorptivity exceeding 90%, relative absorption bandwidth, thickness, and flexibility. Detailed comparison results are presented in Table 2. The results indicate that the proposed metamaterial wave absorber exhibits a broad absorption frequency band, outstanding conformal and optically transparent physical properties, as well as a compact size and thickness. These characteristics underscore its significant potential for applications in stealth technology.

This paper presents the design of an absorber that is optically transparent, low-profile, ultra-wideband, and insensitive to polarization and angle. It also ensures high operational efficiency as well as microwave and millimeter-wave stealth when applied to solar panels. This research has achieved a significant collaborative breakthrough in three key characteristics: “ultra-wideband absorption (97.11 GHz),” “optical transparency,” and “flexible conformality.” These advancements address industry challenges related to multi-spectral band compatibility and dynamic deployment adaptability in satellite stealth.

## Conclusion

A novel wideband flexible transparent metamaterial absorber, utilizing an ITO conductive film, is proposed in this paper. The absorber employs flexible PVC as the dielectric substrate, while PET coated with an ITO conductive film serves as both the reflective base plate and the resonant layer. In this paper, we present the design of the ITO conductive film pattern for the resonant layer, which achieves an absorption rate exceeding 90% within the frequency range of 11.09 GHz to 108.20 GHz. Furthermore, the relative absorption bandwidth can reach as high as 162.81%. The resonant unit of the absorber exhibits a pattern characterized by central symmetry, which endows it with remarkable polarization-insensitive properties. Notably, the absorption rate remains above 75% even when the angle of oblique incidence reaches 45°, thereby satisfying the requirements for most applications. In this paper, we present a novel absorber that demonstrates excellent absorption performance even under conformal conditions. Our designed ultrathin, broadband, polarization- and angle-insensitive optically transparent metamaterial absorber can not only be applied to microwave stealth design for satellite solar panels but also holds potential for deployment on critical aircraft components such as canopies and radomes.

## Data Availability

All data required to evaluate the findings of this work is available in the presented paper. Additional data related to this work may be requested from the corresponding author.

## Materials availability

CST Studio Suite (version no. 2021.01, https://www.3ds.com), Origin (version no. 2019b, https://www.originlab.com), 3Ds Max (version no. 2014, https://www.autodesk.com).
